# Weakly Polarized Organic Cation-Modified Hydrated Vanadium Oxides for High-Energy Efficiency Aqueous Zinc-Ion Batteries

**DOI:** 10.1007/s40820-024-01339-y

**Published:** 2024-02-22

**Authors:** Xiaoxiao Jia, Chaofeng Liu, Zhi Wang, Di Huang, Guozhong Cao

**Affiliations:** 1 Department of Materials Science and Engineering, University of Washington, Seattle, WA 98195 USA; 2 School of Materials Science and Engineering, Tongji University, Shanghai, 201804 People’s Republic of China

**Keywords:** Zinc-ion battery, Vanadium oxide, V_2_O_5_·*n*H_2_O, Pre-intercalation, Interlayer engineering

## Abstract

Vanadium oxides, particularly hydrated forms like V_2_O_5_·*n*H_2_O (VOH), stand out as promising cathode candidates for aqueous zinc ion batteries due to their adjustable layered structure, unique electronic characteristics, and high theoretical capacities. However, challenges such as vanadium dissolution, sluggish Zn^2+^ diffusion kinetics, and low operating voltage still hinder their direct application. In this study, we present a novel vanadium oxide ([C_6_H_6_N(CH_3_)_3_]_1.08_V_8_O_20_·0.06H_2_O, TMPA-VOH), developed by pre-inserting trimethylphenylammonium (TMPA^+^) cations into VOH. The incorporation of weakly polarized organic cations capitalizes on both ionic pre-intercalation and molecular pre-intercalation effects, resulting in a phase and morphology transition, an expansion of the interlayer distance, extrusion of weakly bonded interlayer water, and a substantial increase in V^4+^ content. These modifications synergistically reduce the electrostatic interactions between Zn^2+^ and the V–O lattice, enhancing structural stability and reaction kinetics during cycling. As a result, TMPA-VOH achieves an elevated open circuit voltage and operation voltage, exhibits a large specific capacity (451 mAh g^–1^ at 0.1 A g^–1^) coupled with high energy efficiency (89%), the significantly-reduced battery polarization, and outstanding rate capability and cycling stability. The concept introduced in this study holds great promise for the development of high-performance oxide-based energy storage materials.

**Supplementary Information:**

The online version contains supplementary material available at 10.1007/s40820-024-01339-y.

## Highlights

- A vanadium oxide (TMPA-VOH) is synthesized with trimethylphenylammonium cations chemically pre-inserted into hydrated vanadium oxide.
- The pre-intercalation of weakly polarized organic cations strategically utilizes both ionic and molecular pre-intercalation effects.
- TMPA-VOH, with modified crystal structure and morphology, increased V^4+^ content, and weakened electrostatic interactions between Zn^2+^ and the V-O lattice, demonstrates enhanced voltage, storage capacity, structural stability, and reaction kinetics.

**Supplementary Information:**

The online version contains supplementary material available at 10.1007/s40820-024-01339-y.

## Graphical abstract

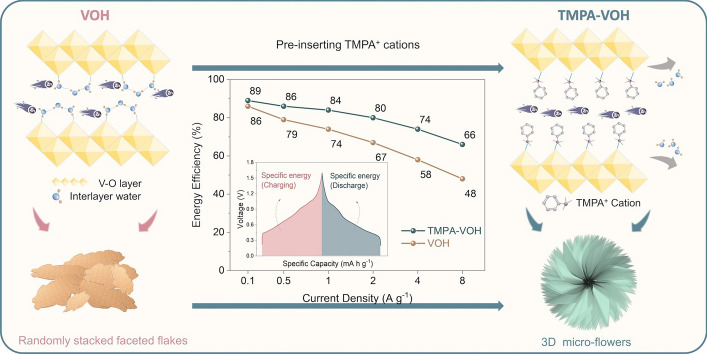

## Introduction

The growing demand for the integration of renewable energy sources into the power grid has emphasized the necessity for safe and cost-effective electrochemical energy storage systems. Although lithium-ion batteries (LIBs) currently dominated the market as the most mature and successfully commercialized technology [1, 2], there is a rising interest in developing alternative technologies to address the requirements for cost reduction and environmental sustainability [3–5]. In this context, aqueous zinc-ion batteries (ZIBs), benefiting from the use of air-stable zinc metal anodes and nonflammable water-based electrolytes, stand out for grid scale and stationary energy storage applications [6, 7]. Among the various potential cathode materials for aqueous ZIBs [6, 8–12], transition metal oxides, particularly vanadium-based compounds, have received increased attention, attributed to their versatile range of oxidation states, adjustable layered crystal structure, and an impressive theoretical capacity of 589 mAh g^−1^ (with a two-electron transfer). Notably, hydrated vanadium oxide V_2_O_5_·*n*H_2_O (VOH) has emerged as particularly promising, featuring an expanded interlayer spacing of approximately 11 Å, mixed valence states (V^5+^ and V^4+^), and facile ion-exchange capability [13, 14]. Despite these advancements, VOH cathodes in aqueous ZIBs exhibit an observable capacity decline, attributed to the gradual loss of stacking order in the V–O layers over prolonged cycling [15].

Another challenge arises from the intrinsic nature of Zn^2+^ ion storage. Despite the small ionic radius of Zn^2+^ (0.74 Å), they typically exists in the solvated form of [Zn(H_2_O)_6_]^2+^ (4.3 Å) in mild aqueous electrolytes [10, 16, 17]. The compact size and high charge density of divalent Zn^2+^ cations pose inherent kinetics challenges, including big charge transfer resistance and sluggish diffusion across the electrode interface due to high de-solvation energy barrier. Additionally, challenges include insufficient capacity resulting from the inadequate utilization of diffusion depth, and cycle life deterioration due to the distortion/collapse of the structure caused by strong electrostatic interactions between Zn^2+^ and the host lattice [18]. Therefore, a significant need persists for the development of vanadium-based cathodes with a more robust framework, enabling faster, deeper, more stable, and more reversible ion intercalation/de-intercalation.

Strategies, including defect engineering, nanosizing, doping, and surface coating, have been developed to optimize the performance of V-based cathodes [6]. Among them, pre-intercalation of foreign species is by far the most effective and facile one, for not only the vanadium oxides but also the manganese oxides and other tunnel or layer structured material [13, 19]. Guest species, including both ionic (main group or transition group ions) [13, 14, 20–22] and molecular species (organic molecules) [24], can undergo pre-intercalation into the tunnels or layers of a material, interacting with the host lattice and charge carriers through chemical bonding, electrostatic interactions, or coordination [23, 24]. Generally, the effects of pre-intercalation involve the regulation of interlayer spacing, enhancement of structural stability, and improvement of electrochemical kinetics. However, various pre-intercalated guest species exhibit fundamental distinctions, influencing the host structure and its energy storage properties differently. For instance, main group metal ions with low electronegativity form robust bonds with the host lattice, effectively promoting structural stability [24]. Transition metal ions, featuring tunable *d* orbitals, interact with the V 3*d* orbital, shifting the redox couple to a higher level and significantly improving open circuit voltage and operation voltage [24, 25]. Organic molecules contribute to a more expanded interlayer distance (exceeding 13 Å) and effectively alleviate electrostatic interactions between Zn^2+^ and the host structure, favoring the ionic diffusion kinetics and stability upon cycling [24, 26, 27]. Considering these factors, an intriguing direction is pre-intercalating the low polarity organic cations into the vanadium oxides, potentially harnessing the advantages of both ionic and molecular pre-intercalation. The larger size of organic cations can further expand the interlayer spacing, surpassing the limits achievable with metal cations. The ionic nature of organic cations enables stronger bondings with the host compared to neutral organic molecules. The low polarity has the potential to alleviate their electrostatic interactions with cycled ions during charging/discharging.

In this study, we designed an organic cation-preintercalated vanadium oxide ([C_6_H_6_N(CH_3_)_3_]_1.08_V_8_O_20_·0.06H_2_O, referred to as TMPA-VOH) by introducing the organic salt trimethylphenylammonium chloride into the V_2_O_5_ precursor during hydrothermal synthesis. The pre-insertion of weakly polarized TMPA^+^ cations into the V–O lattice induces notable changes in phase and morphology, enlarges the interlayer distance (from 11.7 to 13.9 Å), increases the V^4+^ content (from 6.8% to 21.1%), and expels the interlayer water molecules (from 1.21 to 0.06 H_2_O). These synergistic modifications result in the weakened electrostatic interactions between Zn^2+^ and lattice, enhanced electronic conductivity, facilitated Zn^2+^ diffusion, and promoted accessibility to additional reactive sites. Capitalizing on these effects, when employed as cathodes in aqueous ZIBs, TMPA-VOH exhibits enhanced capacitive behavior, reduced battery polarization, and demonstrates significant performance metrics, including a high open circuit voltage of 1.58 V, a large specific capacity of 451 mAh g^–1^ with a high energy efficiency of 89% (at 0.1 A g^–1^), superior rate performance (a capacity of 294 mAh g^–1^ at 8 A g^–1^), and prolonged cycling stability (87% capacity retention after 2000 cycles).

## Experimental Section

### Materials Preparation

TMPA-VOH was synthesized via a hydrothermal method. 2 mmol of V_2_O_5_ and 2 mL of H_2_O_2_ (30 wt%) were added to 50 mL of deionized water. In a separate beaker, 1 mmol of trimethylphenylammonium chloride was dissolved in 30 mL of deionized water. The two solutions were then mixed and stirred for 30 min. The resulting solution was transferred to a 100 mL Teflon-lined autoclave and maintained at 120 °C for 6 h. Then the precipitates were collected by centrifugation and washed with deionized water and ethanol. The precipitates were first air-dried at 70 °C overnight and then vacuum dried at 120 °C for 2 h. For comparison, VOH was synthesized using the same procedure, excluding the addition of the organic salt and was dried using a freeze dryer.

### Material Characterizations

Phase identification was carried out using a powder X-ray diffractometer (XRD, Bruker D8 Discover). Microstructural, morphological, and elemental analyses were conducted using FEI Sirion XL30 scanning electron microscope (SEM) equipped with the Oxford Instruments Energy Dispersive X-ray Spectrometer (EDS) system, and an FEI Tecnai G^2^ F20 SuperTwin 200 keV Transmission Electron Microscope (TEM) equipped with an EDAX Elite-T EDS. The surface chemical composition was quantified using a Kratos Axis Ultra DLD X-ray photoelectron spectroscopy (XPS) with a monochromatic Al Kα x-ray source. Thermal stability and water content were measured using a dual thermogravimetric analysis/differential scanning calorimetry instrument (Mettler Toledo TGA/DSC 3 +). Functional groups and chemical bonds were identified by a Fourier transform infrared (FTIR) spectroscopy (Thermo Scientific Nicolet iS10) with laser excitation at 514 nm. Electron paramagnetic resonance (EPR) tests were conducted using a Bruker A300 spectroscopy.

### Electrochemical Measurements

The TMPA-VOH and VOH cathodes were prepared as follows: first, active materials, carbon black, and polyvinylidene fluoride were blended in a weight ratio of 7:2:1 and dispersed in N-Methyl-2-Pyrrolidone solvent to form a uniform slurry. Then, the slurry was coated onto a titanium foil and dried at 60 °C, followed by additional drying in a vacuum oven at 120 °C for 12 h. Electrochemical tests were conducted using CR2032 coin cells, with metallic zinc serving as the counter electrode, glass fiber as the separator, and a 3 M zinc trifluoromethanesulfonate (Zn(CF_3_SO_3_)_2_) aqueous solution as the electrolyte. All cells were assembled in air and tested within a voltage range of 0.2–1.6 V (*vs.* Zn^2+^/Zn). Galvanostatic charge and discharge (GCD) tests were performed using the Neware Battery Testing Systems (CT-4008). Cyclic voltammetry (CV) and electrochemical impedance spectroscopy (EIS) data were collected on the Solartron SI 1287 Potentiostat and Galvanostat equipped with a 1260 Solartron Impedance Analyzer.

## Results and Discussion

### Morphology and Structure

**Fig. 1:**
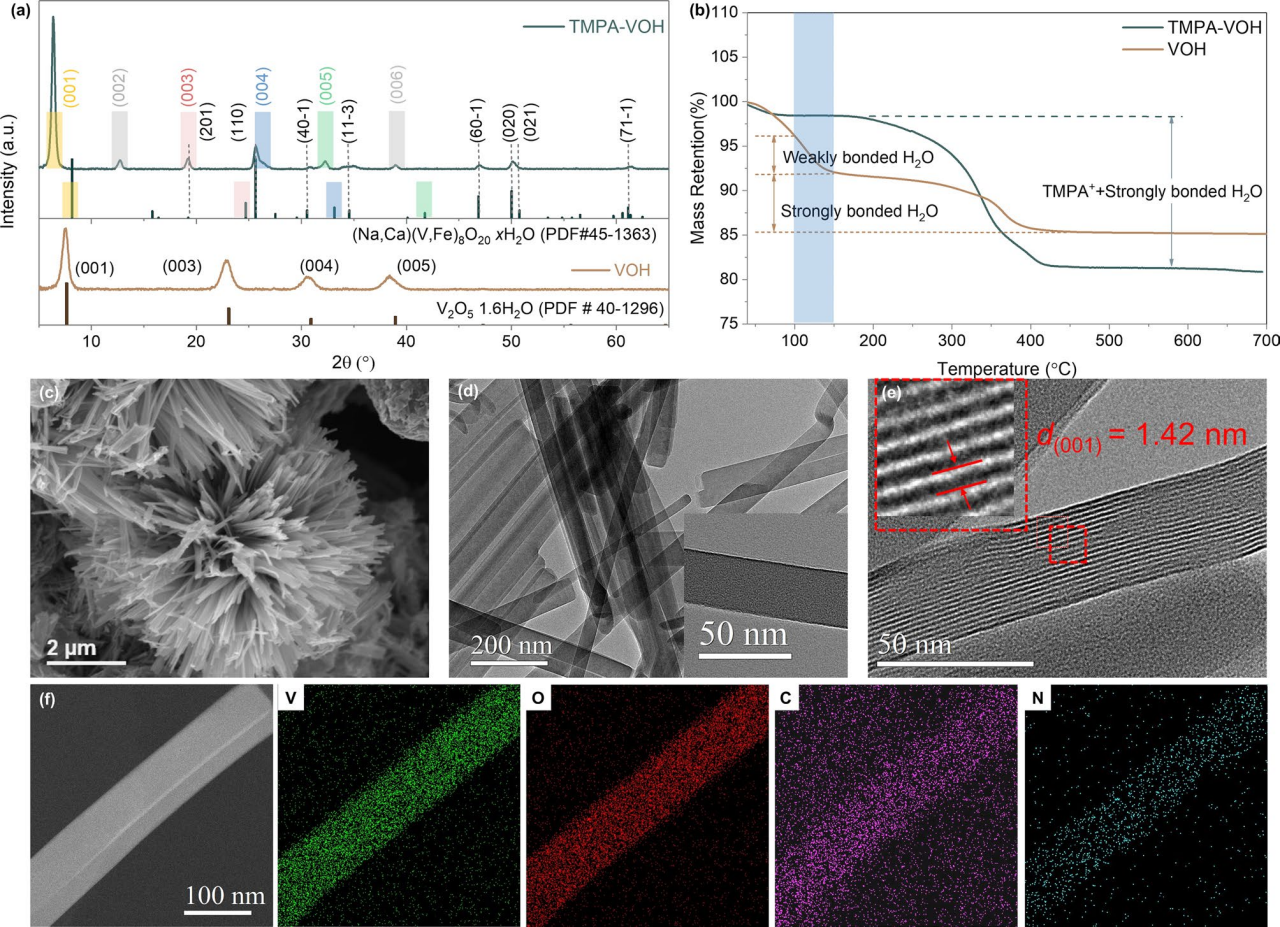
Structural and morphological characterization of TMPA-VOH and VOH*.* **a **XRD patterns. **b** TG curves. **c** SEM image, **d, e** TEM images and **f** the corresponding EDS elemental mappings of TMPA-VOH

TMPA-VOH is synthesized using the same hydrothermal conditions as VOH, with the addition of trimethylphenylammonium chloride (TMPACl) salts at a V_2_O_5_/TMPACl ratio of 2. The formation of dark green precipitates implies a significant reduction of V^5+^ to V^4+^ [28]. Figure 1a illustrates the X-ray diffraction (XRD) patterns of TMPA-VOH and VOH. VOH exhibits a typical layered structure with characteristic peaks (001), (003), (004), and (005), well-matched to the V_2_O_5_·1.6H_2_O pattern (JCPDS No. 40–1296) [14]. The main (001) peak at 2θ of 7.54° corresponds to an interlayer distance of approximately 11.7 Å in VOH. However, upon introduction of TMPA^+^ cations, the diffraction peaks of TMPA-VOH align with those of the monoclinic (Na, Ca)(V, Fe)_8_O_20_·*n*H_2_O phase (Space group: C2/m, JCPDS No. 45–1363) [29]. As clearly depicted in Fig. 1a, all the (00*l*) peaks of TMPA-VOH shift to a lower degree compared to the standard (Na, Ca)(V, Fe)_8_O_20_·*n*H_2_O phase. Meanwhile, all the other peaks such as (201), (110), (40 $\overline{1 }$), (11 $\overline{3 }$), (60 $\overline{1 }$), (020), (021), and (71 $\overline{1 }$) remain at the same positions, strongly indicating that TMPA^+^ has entered the interplanar space of the V–O structure. Compared to the standard (Na, Ca)(V, Fe)_8_O_20_·*n*H_2_O pattern, the (00l) peak of TMPA-VOH shifts from 8.18° to 6.36°, corresponding to an expansion of lattice spacing from 10.8 to 13.9 Å. A schematic illustration of the TMPA-VOH structure is provided in Fig. S1. The [V_8_O_20_] layer is composed of four-fold [V_4_O_10_] chains, formed by (opposite) corner sharing [VO_6_] octahedra (parallel to *b*). Those chains densely packed by sharing edges to form layers, with sufficient interlayer sites partially occupied by cations or water molecules [29].

Scanning electron microscopy (SEM) images (Fig. 1c) and transmission electron microscopy (TEM) images (Fig. S2a) reveal that TMPA-VOH exhibits a three-dimensional flower-like/urchin-like morphology, comprising numerous long and ultrathin VO_x_ nanobelts. These nanobelts nucleate from the core and grow radially outward in all directions, typically reaching several micrometers in length and less than 100 nm in width (Figs. 1d and S2b, c). This suggests a rapid diffusion-controlled crystal growth without secondary nucleation. The high-resolution transmission electron microscopy (HRTEM) image of TMPA-VOH (Figs. 1e and S2d) displays lattice fringes with an interlayer spacing of 1.42 nm, corresponding to the *d*-spacing of (001) planes from the XRD pattern (Fig. 1a), implying a preferential growth direction for the nanobelts. In contrast, VOH exhibits a more agglomerated and irregular morphology (Fig. S3), consisting of randomly stacked faceted flakes. This difference implies that the incorporation of TMPA^+^ cation or the presence of TMPACl induces a significant morphology change.

Possible explanations for this morphological change might be: the uniformly distributed TMPA^+^ cations in the V_2_O_5_ sol could (1) provide more initial nucleation sites, (2) alter the surface energy of nuclei and crystals by adsorbing TMPA^+^ cations onto the surface, (3) lower the critical energy barrier of initial nucleation and/or subsequent crystal growth, and (4) slow the supply or diffusion of vanadium oxide growing species to the growth front. These factors lead to the formation of a morphology far from the thermodynamic equilibrium shape. The 3D micro flower-like morphology with ultrathin nanobelts of TMPA-VOH provides a larger accessible surface area for ion diffusion and more open space for electrolyte penetration, reducing the diffusion pathways for guest ions. TEM energy dispersive spectrum (TEM-EDS) element mapping (Fig. 1f) and SEM–EDS (Fig. S4) confirm the uniform distribution of C, N, V, and O throughout the entire TMPA-VOH architecture, validating the successful introduction of TMPA^+^ into the V–O lattice. The molar ratio of N:V, calculated to be ~ 13.6% based on the SEM–EDS and TEM-EDS, gives an average stoichiometric formula of (TMPA)_1.08_V_8_O_20_·*n*H_2_O for TMPA-VOH.

The water content in TMPA-VOH and VOH is determined through thermogravimetric (TG) analysis. In Fig. 1b, the water loss in VOH is divided into: (1) a first stage (< 150 °C) corresponding to the loss of weakly bonded water molecules (reversibly adsorbed between the V–O layers), and (2) subsequent stages (150–450 °C) corresponding to the removal of more tightly bonded water within the layers [30]. The stoichiometric formula of the prepared VOH was determined to be V_2_O_5_·1.21H_2_O, with a total mass loss of 10.4% between 100 to 450 °C. Notably, in TMPA-VOH, there is a negligible mass loss in the first stage (below 150 °C), indicating the absence of reversibly adsorbed water molecules between layers. It can be inferred that intercalating TMPA^+^ cations into the V–O ribbons is accompanied by the expulsion of interlayer water molecules. The primary weight loss of TMPA-VOH (~ 17%) occurs between 150 and 450 °C, attributed to the removal of strongly bonded crystal water and the combustion of organic components in air. Based on the N content from EDS data (N: V ~ 13.6%), the amount of TMPA^+^ cation and crystal water can be estimated, determining the formula of TMPA-VOH roughly to be (TMPA)_1.08_V_8_O_20_·0.06H_2_O.

The combined results of XRD, SEM/TEM and TG affirm that the intercalation of large, weakly polarized organic TMPA^+^ cations induces a phase change from V_2_O_5_·*n*H_2_O to (TMPA)_1.08_V_8_O_20_·*n*H_2_O. This transformation is characterized by an interlayer spacing expansion from 11.7 to 13.9 Å, a morphology change from randomly stacked faceted flakes to 3D spherical micro-urchins, and the extrusion of weakly bonded water molecules between interlayers.

**Fig. 2:**
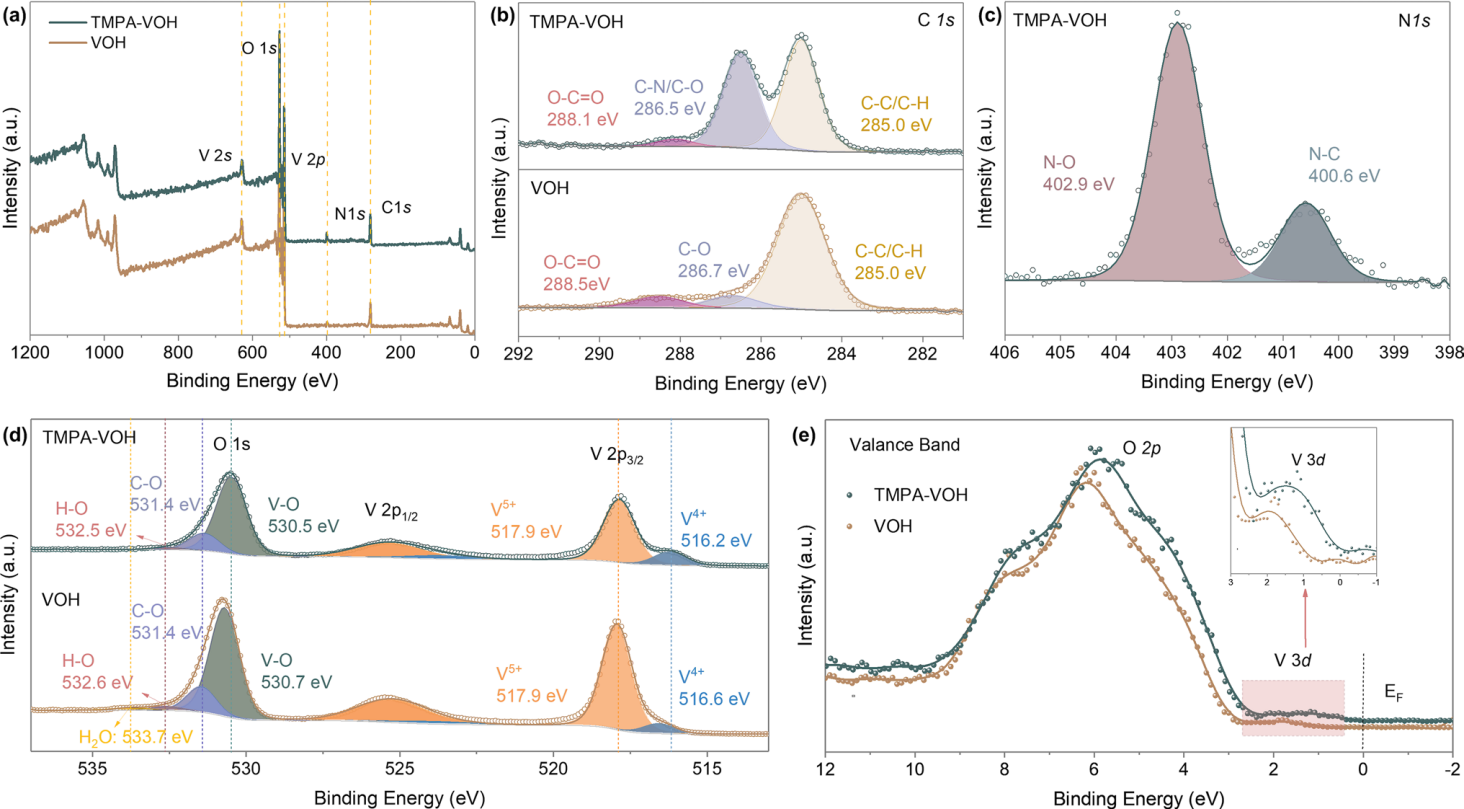
XPS analysis of TMPA-VOH and VOH. **a** XPS survey spectra, showing a clear N signal but no Cl signal in TMPA-VOH. High resolution of **b** C 1*s* spectra, a strong C–N peak in TMPA-VOH confirm the insertion of TMPA^+^ cations. **c** N 1*s* spectra, indicating the presence of C–N bonds and the formation of N–O bonds in TMPA-VOH. **d** V 2*p* and O 1*s* spectra, illustrating the introduction of a large amount of V^4+^ ions and the expulsion of interlayer water by TMPA^+^ pre-insertion. **e** VB spectra of TMPA-VOH and VOH

The chemical composition and valance state of TMPA-VOH are elucidated through XPS. The XPS survey spectra of TMPA-VOH (Fig. 2a) reveal signals from V, O, N, and C, confirming the presence of TMPA^+^ cations in the material. Notably, no trace of Cl signal is detected in the survey spectra (Fig. 2a) or the high-resolution Cl 1*s* spectra (Fig. S5), indicting the exclusive insertion of organic TMPA^+^ cations without Cl^−^ anions. In the high-resolution C 1*s* spectra of VOH (Fig. 2b), in addition to the main line at the binding energy of 285.0 eV (C–C/C–H bonding), two other weak but recognized peaks at 286.7 and 288.5 eV are observed, assigned to the C–O, and O–C = O components [31]. These detectable carbon components in VOH originate from surface contamination, commonly observed in samples exposed to the atmosphere [32, 33]. In comparison to the C 1*s* of carbon contamination in VOH, the C 1*s* spectra of TMPA-VOH (Fig. 2b) reveal a strong resolved peak at 286.5 eV, corresponding to the C–N bonds from the amino group in TMPA^+^. The presence of C–-N bonds or the amino group is also evident at 400.6 eV in the N 1*s* spectra (Fig. 2c), where the other peak at 402.9 eV could be attributed to N-O bonds formed between TMPA^+^ cations and oxygen in the vanadium oxide layer [34, 35]. This further verifies the successful pre-intercalation of TMPA^+^ cations into the VOH lattice and the formation of chemical bonds between nitrogen atoms (from TMPA^+^) and oxygen atoms (on the interlayer surface of vanadium oxide).

The high-resolution V 2*p*_*3/2*_ spectrum of TMPA-VOH (Fig. 2d) reveals a prominent peak at 517.9 eV and a shoulder at 516.2 eV, corresponding to V^5+^ and V^4+^ ions, respectively [31, 36, 37]. From the fitted peak area, the ratio of V^4+^/V^5+^ in TMPA-VOH is calculated to be 21.1%, resulting in an average vanadium valence state of + 4.83 (in agreement with the literature data of 4.8 [29]). In contrast, VOH has a V^4+^/V^5+^ ratio of 6.8%, estimating a vanadium valence state of + 4.94. The substantial increase in the proportion of V^4+^ ions after TMPA^+^ introduction promotes the hopping of unpaired electrons between V^4+^ and V^5+^, enhancing the electronic conductivity and electrochemical kinetics of TMPA-VOH. An intriguing feature from Fig. 2d is the full width at half maximum (FWHM) of the V 2*p*_*3/2*_ peak in TMPA-VOH (1.23 eV), which is broader than that of VOH (1.14 eV). The V 2*p* width of vanadium oxides depends on the population of the *d* band; VO_x_ with more unpaired electrons exhibits a broader line [36, 38, 39]. The broadening of the V 2*p* line for TMPA-VOH suggests a higher concentration of electrons in the *d*-band, further illustrating the significant increase in V^4+^ ions after the introduction of TMPA^+^. The O 1*s* spectra of TMPA-VOH were deconvolved into three components at 530.5, 531.4, and 532.5 eV (Fig. 2d). The dominant peak at 530.5 eV arises from the V–O lattice [36, 37]. The latter two components can be assigned to C–O and H–O groups, coming from the surface contamination on the sample [31, 36], as also detected in the C 1*s* spectra (Fig. 2b) [37]. In the O 1*s* region of VOH, an additional peak at 533.7 eV is observed, related to the interlayer water molecules [31, 38]. Notably, the corresponding peak is absent in TMPA-VOH, suggesting that the introduction of TMPA^+^ cations not only intercalate into the interlayers but also expels the interlayer water molecules, consistent with the TG results.

The valence band (VB) spectrum of TMPA-VOH and VOH is presented in Fig. 2e. Both samples exhibit a dominated band at ~ 6 eV (from O 2*p* contribution) and a low intensity band at ~ 1.5 eV (from V 3*d* contribution) [36]. The O 2*p* component is associated with the emission from the vanadyl oxygen (center peak), chain oxygen, and bridge oxygen in the VO_x_ structure, hybridized with V 3*d*. The appearance of the V 3*d* component, which will not be observed in the orthorhombic V_2_O_5_, confirms the presence of V^4+^, attributed to its higher electron density in the conduction band [36, 40]. In comparison with VOH, the valance band of TMPA-VOH exhibits a slight shift to lower energy. This indicates increased V–O interactions in TMPA-VOH, possibly arising from the interaction of TMPA^+^ with the VO_x_ interlayer surface, resulting in a subtle change in V–O bond length or angle [41].

**Fig. 3:**
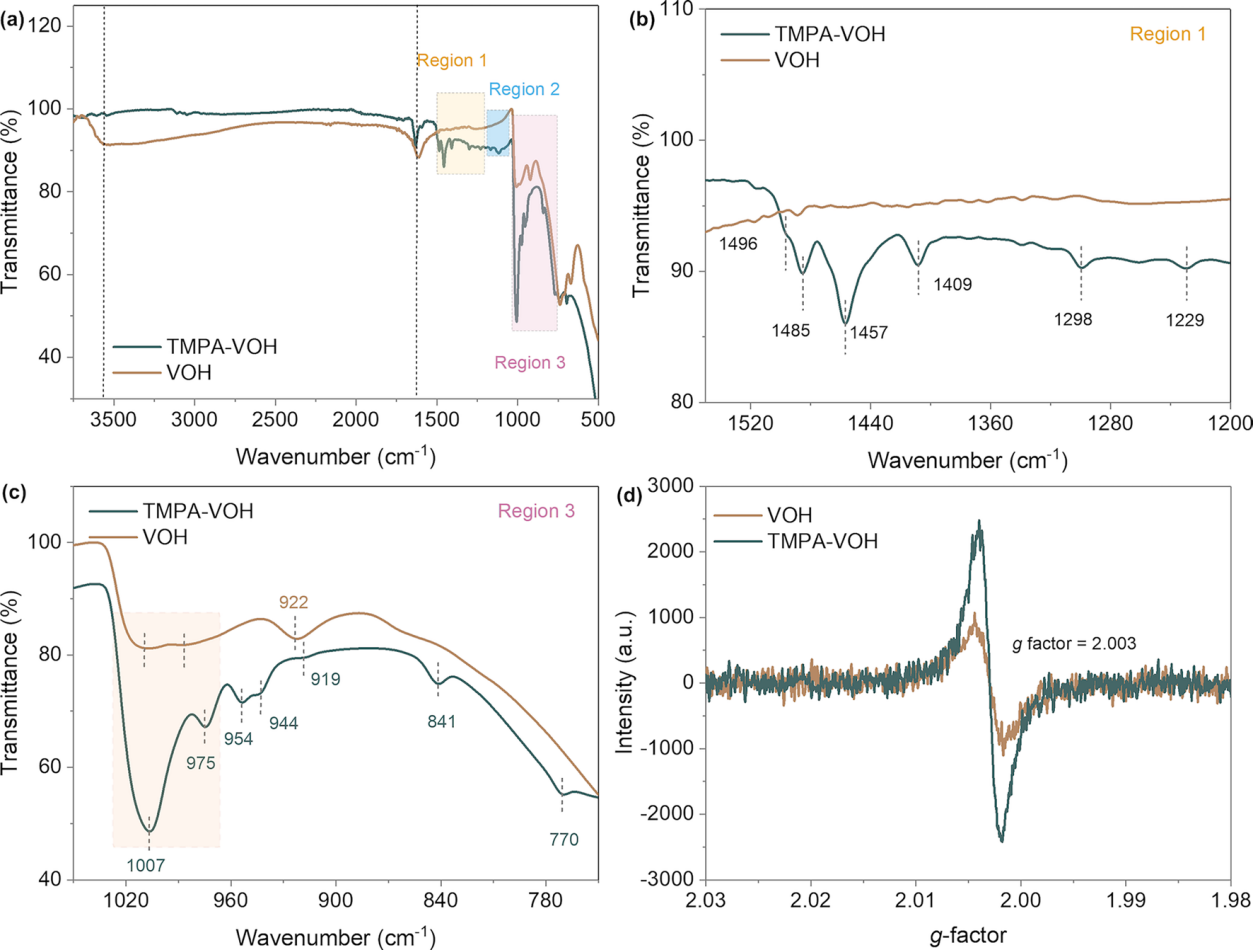
Chemical interactions characterization by FTIR and EPR results. **a** FTIR spectra of TMPA-VOH and VOH. **b** FTIR spectra in the region of 1550–1200 cm^−1^ show the characteristic vibration modes of TMPA^+^ species in TMPA-VOH. **c** FTIR spectra in the region of 1050–750 cm^−1^ depict the splitting of the V = O peak and the absence of interlayer water in TMPA-VOH. **d** EPR spectra of TMPA-VOH and VOH show a stronger signal of unpaired electrons in TMPA-VOH, confirming the presence of more V^4+^ species

Figure 3 displays the FTIR spectra of TMPA-VOH and VOH, with corresponding frequencies and assignments listed in Table S1. In the TMPA-VOH spectra, distinctive peaks in the 1550–1200 cm^−1^ region (Region 1 in Fig. 3a, b) and 1030–1180 cm^−1^ region (Region 2 in Fig. 3a and Fig. S6) correspond to characteristic vibration modes of –N–CH_3_, –CH_3_, and the C–H ring in the TMPA^+^ salt [42], affirming the presence of TMPA^+^ in the TMPA-VOH lattice. In Fig. 3a, c, peaks observed at 742, 975, and 1007 cm^−1^ in TMPA-VOH represent the characteristic bands of the vanadium oxide framework [43, 44]. The 738 cm^−1^ peak in VOH is related to the asymmetric stretching of V–O–V bridging bands, with the shift to a higher wavenumber in TMPA-VOH (742 cm^−1^) indicating a stronger V–O–V bond in TMPA-VOH [43]. The symmetric stretching of V–O–V observed at 586 cm^−1^ in VOH is not detected in TMPA-VOH [45]. In the enlarged spectra (Region 3 in Fig. 3a, c), the broad peak near ~ 1010 cm^−1^ in VOH stems from the symmetric stretching of the terminal oxygen V = O bond. In TMPA-VOH, this V = O bond splits into two peaks at 1007 and 975cm^−1^, possibly corresponding to the stretching vibrations of V^5+^ = O and V^4+^ = O, respectively [43, 44, 46, 47]. The higher concentration of V^4+^ ions in TMPA-VOH (V^4+^/V^5+^ ratio of 21.1%) results in a significant difference between the two signals, leading to a pronounced splitting of the V = O peak. In contrast, VOH has a lower proportion of V^4+^ ions (V^4+^/V^5+^ ratio of 6.8%), making the splitting of the V = O peak less obvious. This phenomenon of separated V = O peaks has been observed in other systems, such as pyridine-V_2_O_5_·*n*H_2_O and Nitrogrn-doped V_2_O_5_, where the authors attributed it to the stronger interactions between PyH^+^ and V = O and the V = O–H_4_N^+^, respectively [48, 49]. In the spectrum of VOH, peaks at 3565 and 1613 cm^−1^ are related to the stretching of O–H and bending of H–O–H in the structural water molecules, respectively [43, 47]. Notably, similar peaks are not well recognized in TMPA-VOH, suggesting the expulsion of most interlayer water upon TMPA^+^ pre-intercalation, in agreement with the TG results.

Electron paramagnetic resonance (EPR) spectroscopy was employed to provide direct evidence for the existence of paramagnetic V^4+^ species, whose outer shell electronic structure 3*d*^1^ has one unpaired electron to interact with the magnetic field. The EPR spectra of TMPA-VOH exhibit a prominent peak centered at a *g*-factor of 1.9728 (with a peak-to-peak line width of ~ 200 G), confirming the existence of V^4+^ ions (Fig. S7) [50]. In Fig. 3d, both TMPA-VOH and VOH display a symmetric signal at a *g* value of 2.003, originating from unpaired electrons captured at the oxygen sites (oxygen free radicals ${{O}_{2}}^{\bullet -}$) [51]. The signal intensity of TMPA-VOH is notably stronger than that of VOH, indicating a higher content of unpaired electrons in TMPA-VOH and emphasizing the formation of more low-valance state V^4+^ ions following TMPA^+^ intercalation.

### Electrochemical Performance

**Fig. 4:**
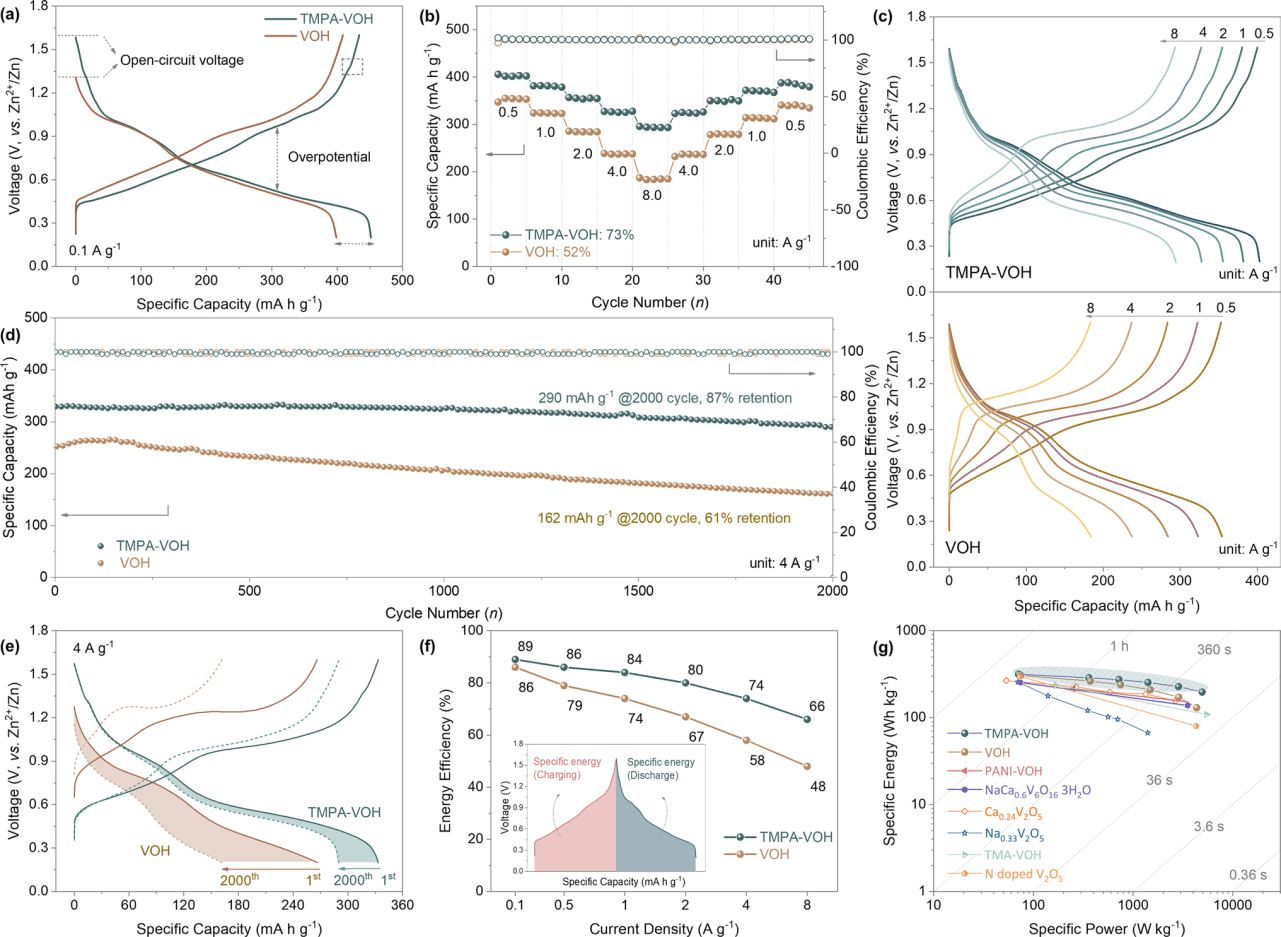
The electrochemical performance of Zn//TMPA-VOH and Zn//VOH cells. **a** The 1st GCD curve at 0.1 A g^–1^. **b** Rate performance and **c** the corresponding GCD profiles at various current densities. **d** Cycling performance at 4 A g^–1^ and **e** GCD profiles of the 1st and 2000th cycle at 4 A g.^–1^. **f** Energy efficiency at various current densities, providing an alternative perspective on rate capability. **g** Ragone plot (specific energy and specific power, based on the weight of the active material in cathode, are derived from the GCD data)

To evaluate the impacts of TMPA^+^ pre-intercalation on Zn^2+^ ion storage properties, we assembled coin cells using TMPA-VOH or VOH as the working electrode, metallic zinc foil as the counter electrode, glass fiber as a separator, and 3 M Zn(CF_3_SO_3_)_2_ as the electrolyte. Figure 4a compares the 1st galvanostatic charge–discharge (GCD) curves of TMPA-VOH and VOH at a current density of 0.1 A g^–1^. TMPA-VOH demonstrates a high open circuit voltage (OCV) of 1.58 V and an initial specific capacity of 451 mAh g^–1^, surpassing that of VOH (OCV: 1.31 V, discharge capacity: 399 mAh g^–1^). Additionally, the mid-point voltage difference of TMPA-VOH is 0.14 V, significantly lower than that of VOH (0.22 V), indicating a reduced discharge/charge overpotential for TMPA-VOH. The rate performance of two samples is illustrated in Fig. 4b. TMPA-VOH delivers discharge capacities of 403, 382, 355, 326, and 294 mAh g^–1^ at current densities of 0.5, 1, 2, 4, and 8 A g^–1^, respectively, demonstrating an outstanding rate performance. In contrast, the discharge capacity of VOH decreases from 354 to 184 mAh g^–1^ with the current density increasing from 0.5 to 8 A g^–1^, resulting in a capacity retention of 52%, much lower than that of TMPA-VOH (73%). The corresponding GCD curves of TMPA-VOH and VOH are depicted in Fig. 4c. The narrower capacity gap between each change in current density and the smaller voltage gap between charge/discharge plateaus (e.g., the mid-point voltage difference at 0.5 A g^–1^ is 0.15 V for TMPA-VOH and 0.27 V for VOH) imply better tolerance to high rates and low polarization of TMPA-VOH, possibly arising from its promoted charge transfer and reaction kinetics.

A comparison of the cycling stability between TMPA-VOH and VOH is present in Fig. 4d. At a current density of 4 A g^–1^, TMPA-VOH exhibits a superior initial discharge capacity of 329 mAh g^–1^, outperforming VOH (264 mAh g^–1^). After 2000 cycles, TMPA-VOH maintains a remarkable capacity of 290 mAh g^–1^, exhibiting a high retention of 87% (of the highest capacity). In contrast, VOH experiences rapid capacity decay, retaining only 162 mAh g^–1^ (61% retention) after cycling. Figure 4e compares the corresponding GCD profiles of the 1st and the 2000th cycle at 4 A g^–1^. Following cycling, the degradation of VOH is evident not only in the reduced specific capacity but also in a significantly enlarged overpotential. Another notable observation from Fig. 4e is that the capacity decay in both samples primarily originates from the lower voltage plateaus (V^4+^/V^3+^), suggesting that the insertion of Zn^2+^ into the low-energy redox sites cannot be fully achieved. This difficulty arises from the gradual collapse of the V–O structure, driven by strong electrostatic interactions between divalent Zn^2+^ ions and the lattice. For TMPA-VOH, the introduction of weakly polarized organic cations serves to widen migration channels and weaken electrostatic interactions, promoting Zn^2+^ diffusion kinetics and preventing obvious structural deterioration and ensuring superior cycling stability.

Energy efficiency, a crucial metric calculated as the ratio of discharge specific energy to charge specific energy, provides a quantifiable measure of the energy consumed or released during the extraction or intercalation of working ions [52]. Charging and discharging at different rates not only impact specific capacity but also influence redox overpotential (Fig. 4c). Therefore, plotting energy efficiency as a function of rates offers a more insightful perspective on the practical rate capability of the electrode. In Fig. 4f, TMPA-VOH consistently demonstrates higher energy efficiency than VOH across all current densities. Notably, as the current density increases from 0.1 to 8 A g^–1^, TMPA-VOH only experiences a moderate drop in energy efficiency, from 89% to 66%, corresponding to an impressive retention of 74%. In contrast, VOH shows a more substantial decay from 86% to 48%, with an energy efficiency retention of 56%.

The superior energy efficiency and its slower decline at higher charge/discharge rates in TMPA-VOH indicate lower overpotential and enhanced rate capability with TMPA^+^ pre-intercalation. This improved reaction kinetics in TMPA-VOH may stem from: (1) its larger diffusion channels, facilitating a lower energy barrier for intercalation ions to reach redox sites; (2) its higher content of low valance state vanadium, providing more unpaired electrons for the conductivity of electroactive materials. Figure 4g presents a Ragone plot comparing the specific energy *vs*. specific power of our Zn//TMPA-VOH and Zn//VOH cells with recently reported aqueous ZIBs. Remarkably, the Zn//TMPA-VOH cell achieves the highest specific energy of 316 Wh kg^–1^ at a specific power of 71 W kg^–1^ and a specific energy of 197 Wh kg^–1^ at a high specific power of 4925 W kg^–1^. These values not only surpass those of the Zn//VOH cell in our study, but also outperform/match many other guest species-intercalated vanadium oxides, including PANI-VOH [53], NaCa_0.6_V_6_O_16_·3H_2_O [54], ([N(CH_3_)_4_]_0.77_,Zn_0.23_)V_8_O_20_·3.8H_2_O [55], N-doped V_2_O_5_ [49], Na_0.33_V_2_O_5_ [56], and Ca_0.24_V_2_O_5_⋅0.83 H_2_O [57].

**Fig. 5:**
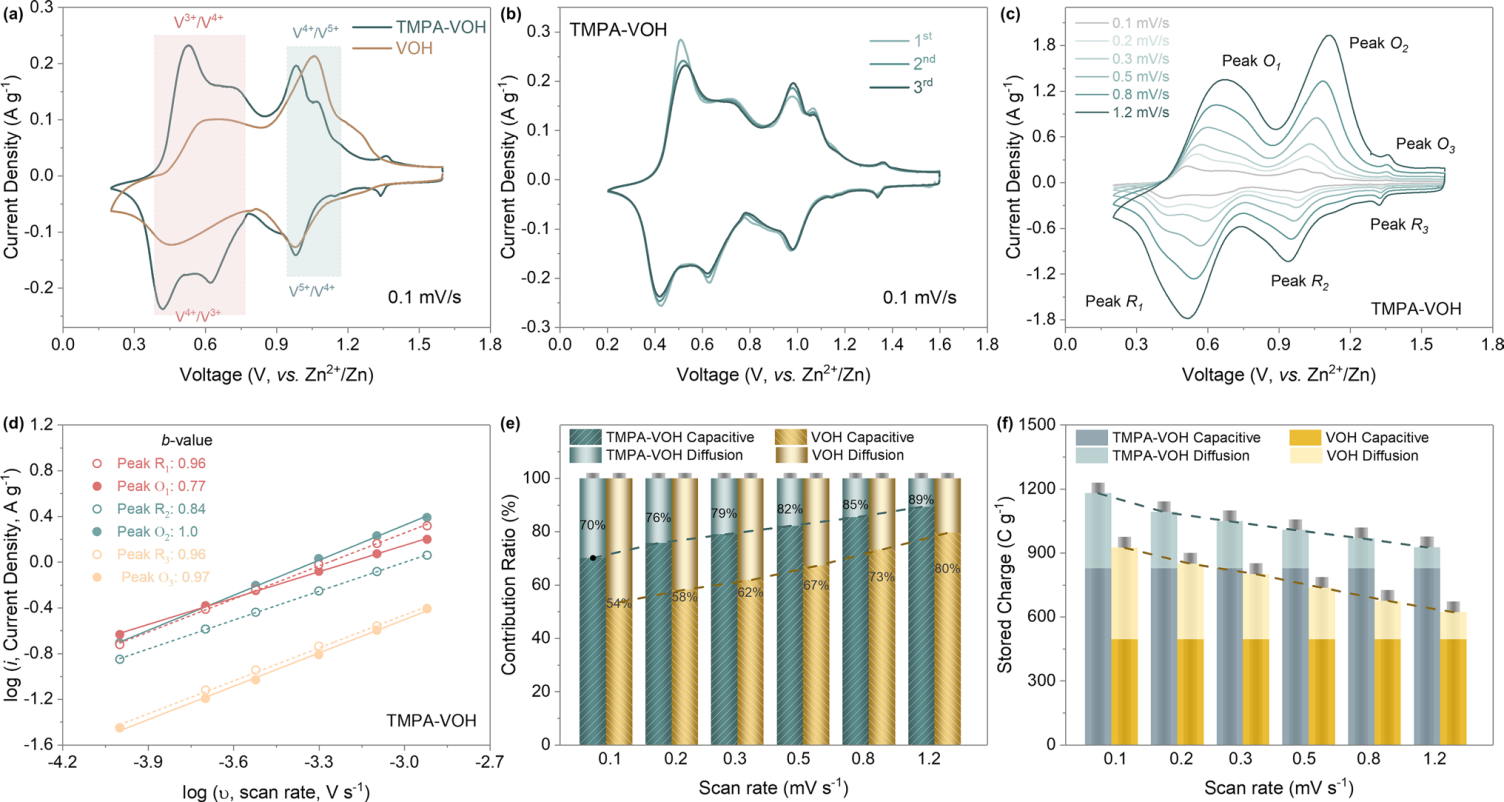
**a** The 3rd cycle of CV tests at a scan rate of 0.1 mV s^−1^. **b** The initial three cycles of CV tests at 0.1 mV s^−1^ for TMPA-VOH. **c** CV curves at various scan rates for TMPA-VOH. **d** The *log* (peak current) *vs. log* (scan rate) plot for each redox peak in TMPA-VOH and their corresponding *b* values. **e** Contribution ratio and **f** specific capacitance of the diffusion-controlled process and capacitive process for TMPA-VOH and VOH at different scan rates

In Fig. 5a, a comparison of cyclic voltammetry (CV) curves at a scan rate of 0.1 mV s^−1^ is presented for TMPA-VOH and VOH. VOH exhibits two distinct pairs of well-defined redox peaks at 0.98/1.06 and 0.45/0.65, representing the redox reactions between V^5+^/V^4+^ and V^4+^/V^3+^, respectively [13, 14, 22]. Surprisingly, TMPA-VOH displays five couples of redox peaks: cathodic peaks centered at 1.33, 0.98, 0.91, 0.61, and 0.43 V, along with respective anodic peaks at 1.35, 0.99, 1.06, 0.67, and 0.51 V. This observation is further supported by the differential capacity (dQ/dV) *vs*. voltage curves at 0.5 A g^−1^ (Fig. S8), revealing five redox couples in TMPA-VOH and aligning well with the CV results. While multiple redox peaks have been reported in various vanadium oxides [58–60], the precise assignment of these peaks remains unclear in the literature.

Firstly, from the initial three CV cycles of TMPA-VOH at 0.1 mV s^−1^ (Fig. 5b), it becomes evident that the five couples of redox peaks in TMPA-VOH remain consistently present and nearly overlapped throughout subsequent cycles. This result strongly suggests the good reversibility of these redox reactions, effectively ruling out the possibility of irreversible phase transitions or structural changes. Secondly, as illustrated in Fig. 5c, with an increase in the scan rate, two pairs of redox peaks around 0.5 V begin to merge into a single pair, so do the two pairs around 1 V. In other words, while TMPA-VOH exhibits five distinct redox pairs at low scan rates, it manifests only three redox pairs at high scan rate. Based on these observations, it is likely that at the small scan rate of 0.1 mV s^−1^, both pairs of redox peaks at 0.98/0.99 V and 0.91/1.06 V can be assigned to the V^5+^/V^4+^ redox reaction, while both pairs at 0.61/0.67 V and 0.43/0.51 V represent the V^4+^/V^3+^ redox centers. This split of vanadium redox peaks could be attributed to the existence of two distinct vanadium coordination in TMPA-VOH. During discharging/charging, the oxidation state of vanadium undergoes transitions between 5 + , 4 + , and 3 + , coinciding with the transformation of the corresponding vanadium coordination polyhedra [58, 61]. In TMPA-VOH, the existence of two distinct polyhedral coordination of vanadium is evident from the separate V = O peaks (V^5+^ = O and V^4+^ = O) in the FTIR spectra (Fig. 3c). Consequently, the transformation of these two types of vanadium coordination occurs at distinct redox voltages, and this becomes more apparent at low scan rates, where the redox reaction is relatively slow. Unlike VOH, where this redox peak split is undistinguishable in CV curves, consistent with the single or nearly merged V = O peaks in FTIR spectra. The additional tiny peak at 1.33/1.35 V suggests the emergence of a new redox site, with some attributing this to the interaction between the empty orbit of Zn^2+^ and the lone pair electrons of N [49].

Further insights can be derived from the CV curves at 0.1 mV s^−1^ (Fig. 5a). The voltage gap between each redox pair of TMPA-VOH is smaller than that of VOH, as detailed in Table S2). This indicates reduced polarization and accelerated reaction kinetics in TMPA-VOH, aligning with its diminished overpotential as evident from the reduced mid-point voltage difference in the GCD curves. The area enclosed by the CV curves corresponds to the charge storage in the material. Notably, the peak area under the V^5+^/V^4+^ peaks is nearly the same for both samples. However, the peak area under the V^4+^/V^3+^ pairs is significantly larger in TMPA-VOH than in VOH. This indicates that TMPA-VOH possesses more accessible redox sites in its structure, facilitating the insertion of more Zn^2+^ ions to reduce vanadium ions to V^3+^. This finding is consistent with the higher specific capacity of TMPA-VOH obtained from the GCD tests.

### Electrochemical Kinetics

CV tests were conducted at various scan rates (ranging from 0.1 to 1.2 mV s^−1^) to reveal the Zn^2+^ ion storage kinetics in TMPA-VOH (Fig. 5c). With increasing scan rates, the voltage difference between each redox pair expands, causing reduction peaks to shift to lower voltage and oxidation peaks to shift to higher voltage. In comparison to VOH (Fig. S9), TMPA-VOH exhibits a less significant enlargement in peak separation, indicating its reduced electrochemical polarization. *b* values, reflective of the dominant charge storage process, were derived from the *log* (peak current) *vs*. *log* (scan rate) plot (Fig. 5d, calculation details in Supplementary Information). For TMPA-VOH, the *b* values of peak O_1_, R_1_, O_2_, R_2_, O_3_, and R_3_ are 0.77, 0.96, 1.0, 0.84, 0.97, and 0.96, respectively. These values suggest that the Zn^2+^ storage in TMPA-VOH involves a combination of diffusion-controlled behaviors and surface-controlled capacitive behaviors [62].

As summarized in Fig. 5e, f, the contribution from each process can be quantitatively distinguished. Firstly, the capacitive contribution in TMPA-VOH at all sweep rates surpasses that in VOH, indicating an overall faster reaction kinetics of TMPA-VOH. This enhanced pseudocapacitive behavior may be attributed to the large surface area and the wide interlayer spacing of TMPA-VOH. Secondly, since capacitive charge storage is sweep rate independent (Fig. 5f), decreases in diffusion-controlled charge storage can serve as an effective indicator for evaluating the rate resistance of electrodes. As shown in Fig. 5e, the contribution ratio of the diffusion-controlled process in TMPA-VOH declines less (from 30 to 11% with scan rate increasing from 0.1 to 1.2 mV s^−1^) than that of VOH (from 46 to 20%), indicating a superior rate capability of TMPA-VOH. Thirdly, from Fig. 5f, the specific capacitance/stored charge in TMPA-VOH (e.g., 1180 C g^−1^ at 0.1 mV s^−1^) surpasses that of VOH (925 C g^−1^ at 0.1 mV s^−1^), consistent with their specific capacify values from GCD tests. Additionally, as scan rates increase, the slower decay of specific capacitance in TMPA-VOH aligns with its improved rate performance (Fig. 4b). In summary, the kinetics analysis from CV data reveals that the charge storage in TMPA-VOH primarily results from the surface-controlled capacitive processes. The pre-intercalation of TMPA^+^ cations enhances pseudocapacitive behavior and results in a slower decay in diffusion-controlled charge storage with increasing scan rate.

**Fig. 6:**
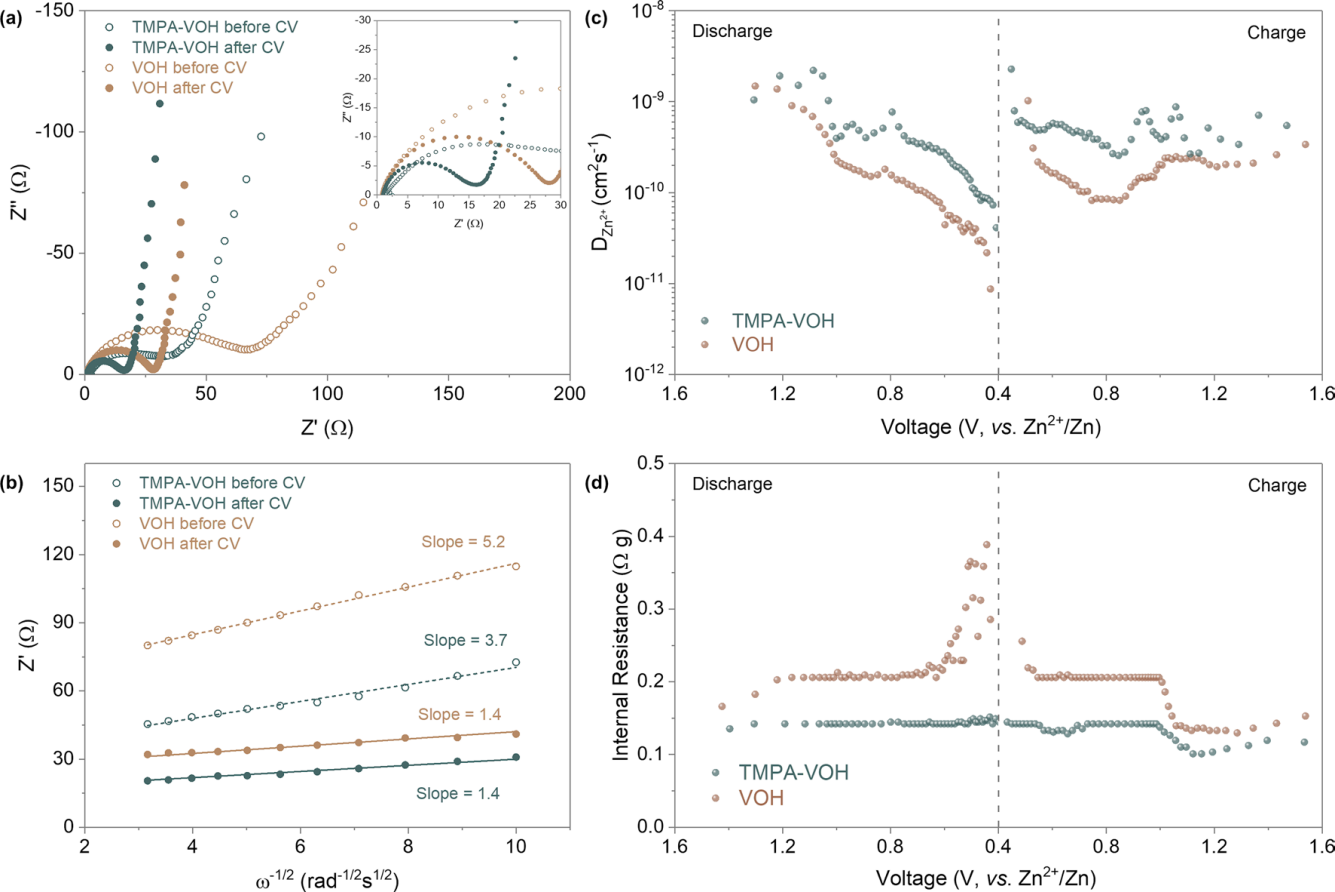
Electrochemical reaction kinetics analysis. **a** Nyquist plots of TMPA-VOH and VOH electrodes before and after CV tests. **b** Plot of the real part of impedance *vs*. ω^−1/2^ in the low-frequency region. **c** Zn ion diffusion coefficients and **d** internal resistance calculated from the 3rd cycle of GITT tests

Figure 6a compares the Nyquist plots of TMPA-VOH and VOH before and after CV tests. Both spectra consist of a semicircle in the high frequency region, associated with the charge transfer resistance (*R*_ct_) in electrodes, and a sloped line at low frequency, related to the ion diffusion process. Before cycling, the *R*_ct_ of TMPA-VOH (28 Ω) is notably half that of the pristine VOH electrode (57 Ω), highlighting improved electronic conductivity resulting from organic cation pre-intercalation. This enhancement is likely attributed to the increased density of unpaired electrons in TMPA-VOH, supported by the significantly higher amount of V^4+^ in TMPA-VOH (21.1%) compared to VOH (6.8%). After cycling, *R*_ct_ decreases for both TMPA-VOH and VOH, reaching 13 and 26 Ω, respectively. The substantial reduction in *R*_ct_ after cycling may be ascribed to the small amount of residual Zn^2+^ in the structure, boosting the electrical conductivity of the cathode. Figure 6b illustrates the real part of impedance (Z’) as a function of frequency (ω^−1/2^) in the low-frequency region. The line for the TMPA-VOH before cycling exhibits a smaller slope than VOH, indicating faster Zn^2+^ ion transport within TMPA-VOH. These EIS results underscore the active role of TMPA^+^ introduction in enhancing the electrochemical reaction kinetics, particularly by boosting charge transfer conductivity.

The Galvanostatic intermittent titration technique (GITT) was employed to compare the ion diffusion kinetics of TMPA-VOH and VOH at various charging/discharging stages. The calculated Zn^2+^ ion diffusion coefficients (D_Zn_^2+^) during the 3rd GITT cycle at 50 mA g^−1^ are presented in Fig. 6c (calculation details in Supplementary Information). TMPA-VOH exhibits D_Zn_^2+^ values ranging from 9.1 × 10^−11^ to 2.4 × 10^−9^ cm^2^ s^−1^ during discharging and 1.9 × 10^−10^ to 2.0 × 10^−9^ cm^2^ s^−1^ during charging. In contrast, VOH displays smaller D_Zn_^2+^ values, approximately 8.6 × 10^−12^ ~ 1.5 × 10^−9^ cm^2^ s^−1^ during discharging and 8.1 × 10^−11^ ~ 1.0 × 10^−9^ cm^2^ s^−1^ during charging. Compared with VOH, the faster Zn^2+^ ion diffusion rate of TMPA-VOH throughout the entire intercalation/de-intercalation process indicates that the pre-insertion of TMPA^+^ into V–O layers effectively benefits the transport of charge carriers. As demonstrated in Fig. 6c, both systems exhibit relatively stable diffusivity at the beginning of the discharge process, followed by a rapid decrease after 1.0 V. Initially, the layered structure with large channels allows the “free” insertion of Zn^2+^ ions; afterwards, deep intercalation is hindered due to the increasing electrostatic interactions between the inserted Zn^2+^. However, this diffusivity decay is more significant in VOH than in TMPA-VOH, especially at lower voltage. This supports the previous statement derived from the CV curves that TMPA-VOH can accommodate more Zn^2+^ ions, inserting into the deeper lattice to reduce more V^5+^/V^4+^ to V^3+^. Another important factor, internal resistance, can be estimated from the IR-drop in the GITT results (Fig. 6d). Internal resistance represents the overall resistance in electrochemical reactions, originating from active materials, current collectors, conductive additives, contact between each element, and electrolyte conductivity. Throughout the charge/discharge process, the internal resistance values for TMPA-VOH are consistently lower than those of VOH (Fig. 6d), contributing to a reduced overpotential and higher energy efficiency for TMPA-VOH, as confirmed by previous GCD and CV results. During discharging (Fig. 6d), the internal resistance of VOH displays two steps of increase at ~ 1.2 and 0.6 V, coinciding with the two-step redox reactions upon the Zn^2+^ intercalation. The dramatic increase in resistance towards the end of discharge in VOH is a consequence of kinetic limitations, attributed to increased concentration polarization and electrochemical polarization. In contrast, the internal resistance of TMPA-VOH remains relatively stable throughout the entire discharge process, further indicating the reduced electrostatic interactions between the inserted Zn^2+^ and the lattice, facilitating the insertion of more Zn^2+^ ions into the TMPA-VOH lattice.

Benefiting from the 3D flower-like morphology, a wide interlayer spacing, and a large amount of V^4+^ with more unoccupied electrons, TMPA-VOH exhibits an accelerated ion diffusion rate and improved charge transfer kinetics. The low polarity of the pre-inserted organic ions minimizes interactions with the cycled Zn^2+^ ions, resulting in reduced polarization. Moreover, the pre-intercalation of organic cations has a significant impact on preventing the decay in diffusion rate and the increase in internal resistance upon deep discharging, enabling relatively stable kinetics throughout the entire discharging/charging process.

### Energy Storage Mechanism

**Fig. 7:**
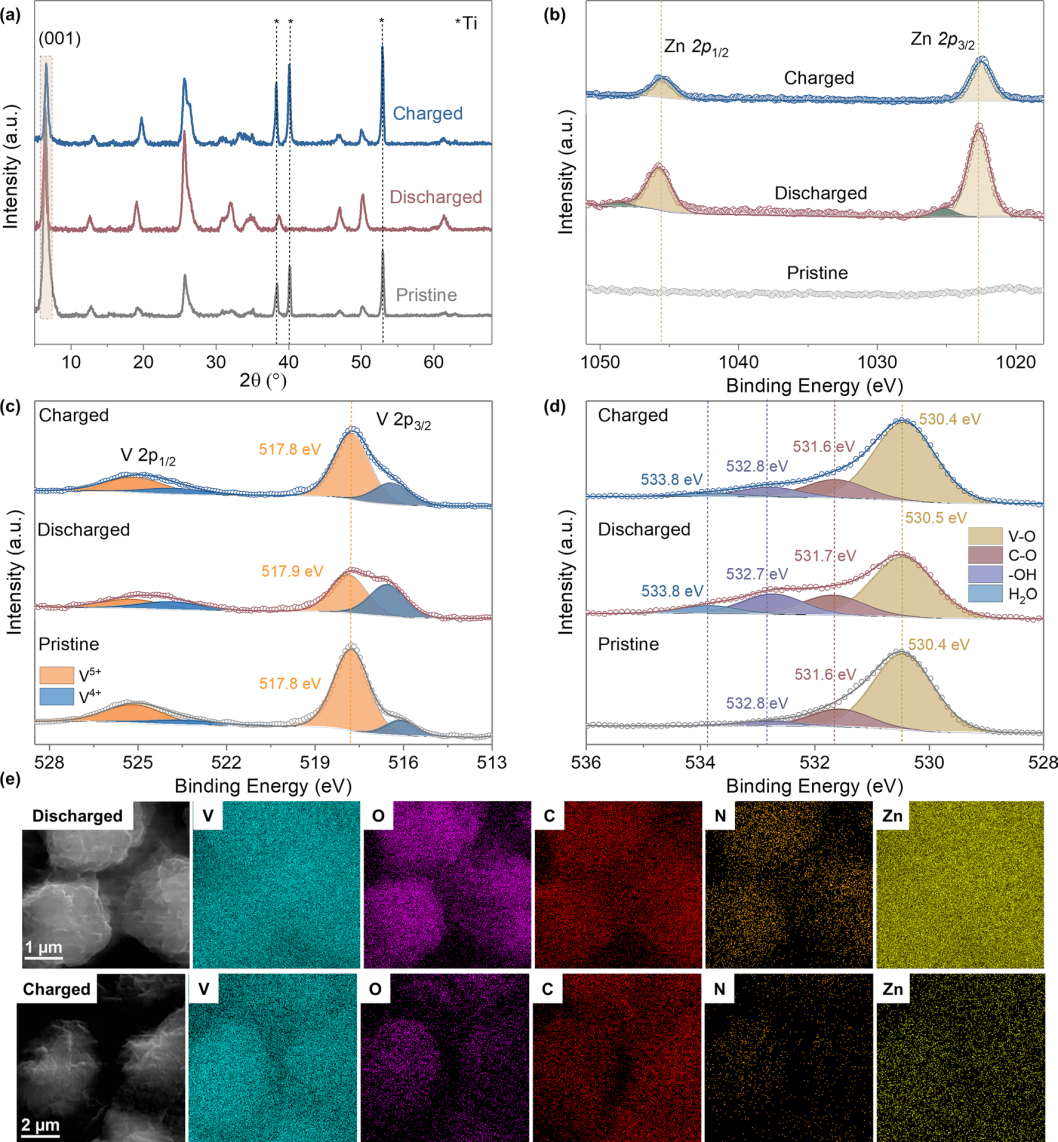
Energy storage mechanism of Zn//TMPA-VOH. **a** Ex situ XRD patterns. Ex situ XPS of **b** Zn 2*p* spectra, **c** V 2*p* spectra, and **d** O 1*s* spectra. **e** SEM elemental mapping images of TMPA-VOH cathode at discharged and charged state

To elucidate the energy storage mechanism of the Zn//TMPA-VOH battery, the structural evolution, valance state and morphological changes of TMPA-VOH during charge/discharge were investigated through ex situ XRD, XPS and SEM analyses. Figure 7a displays the XRD pattern of TMPA-VOH electrodes in the pristine state, fully discharged state (0.2 V) and fully charged state (1.6 V) during the first cycle. The XRD pattern of the pristine electrode closely matches the as synthesized TMPA-VOH powder and the Ti current collector. Upon complete discharge to 0.2 V, there is no significant shift in the position of the (00*l*) peak, indicating minimal expansion or contraction of the interlayer distance during Zn^2+^ intercalation into the structure. This contrasts with some reported vanadium oxides that exhibit noticeable interlayer expansion, which was attributed to screened electrotactic repulsions between V–O layers caused by inserted Zn^2+^ [13]. Conversely, certain vanadium oxides demonstrate an obvious interlayer contraction explained by the electrostatic attractions between Zn^2+^ and the host structure, pulling the layers closer [34, 55, 63]. The negligible interlayer spacing variation in TMPA-VOH suggests alleviated electrostatic interactions between intercalated Zn^2+^ ions and the V–O lattice due to TMPA^+^ pre-insertion [27]. This reduction in electrostatic interactions is beneficial for Zn^2+^diffusion and structural stability during cycling.

It is noteworthy that no discernible new peaks are identified in the discharged electrode, which is different from observations in some studies where the generation of byproducts or secondary phases, such as Zn_x_(CF_3_SO_3_)_y_(OH)_2x-y_·*n*H_2_O or Zn_3_(OH)_2_V_2_O_7_·2H_2_O, is commonly observed during the discharging process [53]. In the case of TMPA-VOH, characteristic peaks of Zn_x_(CF_3_SO_3_)_y_(OH)_2x-y_·*n*H_2_O or Zn_3_(OH)_2_V_2_O_7_·2H_2_O phases cannot be clearly detected, possibly due to the low abundance of new phases generated during discharging, resulting in an XRD signal too weak to be identified [55]. Additionally, the main peaks corresponding to Zn_3_(OH)_2_V_2_O_7_·2H_2_O (JCPDS No. 50–0570) appear at 12.3°, 20.9°, 30.1°, and 34.2° [64], closely located to the peaks of (002), (003), (40 $\overline{1 }$), and (11 $\overline{3 }$) planes of TMPA-VOH (at 12.6°, 19.2°, 30.8°, and 34.7°, respectively), making it even more challenging to differentiate the new phases from the main peaks. However, based on the XPS results, we speculate the formation or disappearance of these new phases upon discharging or charging (as discussed later). This phenomenon is similar to that observed in (Na,Mn)V_8_O_20_·*n*H_2_O [65] and [N(CH_3_)_4_]_0.77_,Zn_0.23_) V_8_O_20_·3.8H_2_O [55].

As shown in the Zn 2*p* XPS spectra (Fig. 7b), no signal is detected for the pristine electrode. When discharged to 0.2 V, strong Zn peaks clearly appear, confirming the intercalation of Zn^2+^ into TMPA-VOH. Upon charged to 1.6 V, the Zn signal is still present but with reduced intensity, suggesting the incomplete extraction of Zn^2+^ from the TMPA-VOH structure. These residual Zn^2+^ ions are believed to be trapped in the host lattice, contributing to enhanced electronic conductivity, as reflected by the reduced charge transfer resistance after cycling (as evidenced by EIS data). Notably, the discharged electrode shows two Zn components in the Zn 2*p*_3/2_ region, a strong one at 1022.7 eV and a small side one at 1025.2 eV, which can be assigned to the intercalated Zn^2+^ in TMPA-VOH and the Zn precipitate (e.g., Zn_x_(CF_3_SO_3_)_y_(OH)_2x-y_·*n*H_2_O or Zn_3_(OH)_2_V_2_O_7_·2H_2_O) generated on the surface [66, 67]. After being fully charged, the latter peak disappears, with only the peak associate with intercalated Zn^2+^ showing at 1022.5 V, suggesting the reversible generation/dissolution of the zinc second phase.

The V 2*p* region is displayed in Fig. 7c. Consistent with the as-synthesized TMPA-VOH material (Fig. 2d), two peaks corresponding to V^5+^ (517.8 eV) and V^4+^ (516.1 eV) exist in the pristine electrode, with V^5+^ being the dominant one. After discharge, the signal of V^4+^ intensifies and becomes the major component, while that of V^5+^ decreases, indicating the reduction of vanadium ions accompanied with the intercalation of Zn^2+^. A shift in the binding energy of V in the discharged state could be attributed to the interactions between intercalated Zn^2+^ and the V–O layers [68]. A smaller shift of the V^5+^ peak in TMPA-VOH (0.1 eV) compared to other guest species pre-intercalated vanadium oxides, like Cr-VOH [69] or Polypyrrole-VOH [70], suggests reduced electrostatic interactions due to TMPA^+^ pre-intercalation. Interestingly, the V^3+^ signal was not observed in the discharged electrode. Although, based on the CV curves or the specific capacity of TMPA-VOH (451 mAh g^–1^), there exists a valence conversion from + 4 to + 3 in vanadium. This phenomenon is widely observed for other vanadium-based cathode [22, 71], as trivalent vanadium cations are very unstable and can be easily oxidized during sample handling (exposed to air) and ex situ XPS characterization. After complete charging, both the position and fraction of vanadium spectra return to the initial state, demonstrating the reversible redox reaction. The slightly higher amount of V^4+^ in the charged electrode than in the pristine electrode can be ascribed to the incomplete de-intercalation of Zn^2+^ in the first charging process, in agreement with the Zn 2*p* spectra analysis (Fig. 7b).

In Fig. 7d, the O 1*s* spectra of the pristine electrode reveal three distinct components at 530.4 V (attributed to lattice oxygen in the V–O matrix), 531.6 V (indicative of C–O from surface contaminations), and 532.8 eV (corresponding to surface O–H species). Upon complete discharge, an additional peak emerges at 533.8 eV, possibly associated with H_2_O molecules. This may be attributed to the insertion of hydrated zinc ions or the formation of Zn_*x*_(CF_3_SO_3_)_*y*_(OH)_2*x*-*y*_·*n*H_2_O or Zn_3_(OH)_2_V_2_O_7_·2H_2_O. The appearance of the H_2_O peak, along with the strengthening of the –OH peak, indicates the possible formation of Zn_*x*_(CF_3_SO_3_)_*y*_(OH)_2*x*-*y*_·*n*H_2_O or Zn_3_(OH)_2_V_2_O_7_·2H_2_O, consistent with the Zn spectra analysis. Upon charging, the diminishing –OH peak and H_2_O peak suggest the decomposition of these newly formed phases. The –OH in the zinc complex arises from the dissociation of water molecules in the electrolyte, induced by the intercalation of H^+^ into the cathode [72]. Therefore, a Zn^2+^/H^+^ co-intercalation mechanism can be reasonably proposed for TMPA-VOH, aligning with many other vanadium oxide cathodes [34, 55, 65]. As demonstrated in the ex situ SEM images (Figs. 7d and S10), there is no significant morphological evolution of TMPA-VOH during charging and discharging. The homogeneous distribution of V, O, N, C, and Zn across the sample further confirms the insertion of Zn^2+^ into TMPA-VOH and the stability of TMPA^+^ cations during discharging/charging. The weak Zn signal detected at the fully charged state suggests the incomplete extraction of Zn^2+^ ions, which agrees with the XPS data. Based on the XRD, XPS, and SEM analyses, the energy storage mechanism of Zn//TMPA-VOH involves a Zn^2+^/H^+^ co-insertion. The pre-intercalation of TMPA^+^ cations significantly decrease the electrostatic interactions between inserted Zn^2+^ and the V–O lattice, resulting in negligible interlayer variation and structural evolution during discharging/charging. This phenomenon is responsible for the enhanced kinetics and stability observed during cycling.

## Conclusions

In this work, weakly polarized organic cations (TMPA^+^) were employed as a novel pre-intercalated species to regulate the structure of vanadium oxide. The resulting [C_6_H_6_N(CH_3_)_3_]_1.08_V_8_O_20_·0.06H_2_O (TMPA-VOH) exhibits enhanced electronic and ionic transports, along with a strengthened structure, attributed to several key factors: (1) the expanded interlayer spacing accelerates Zn^2+^ diffusion and reduces the migration energy barrier; (2) the expelling of interlayer water by the pre-inserted low polarity organic cations alleviates the electrostatic interactions between cycled Zn^2+^ ions and the lattice, minimizing the degradation in both structural stability and ionic diffusivity during cycling; (3) the increased amount of low valence-state V^4+^ provides more unpaired electrons for electronic conductivity; (4) the porous flower-like morphology, constructed from thin nanobelts, shortens ion diffusion pathways and facilitates fast near-surface activities. Capitalizing on these effects, when employed as cathodes in aqueous ZIBs, TMPA-VOH demonstrates enhanced capacitive behavior, reduced battery polarization, and achieves a high open circuit voltage of 1.58 V, a large specific capacity of 451 mAh g^–1^ (at 0.1 A g^–1^) with an energy efficiency of 89%, high rate capability (294 mAh g^–1^ at 8 A g^–1^) and long-term cycling stability with a capacity retention of 87% after 2000 cycles.

## Supplementary Information

Below is the link to the electronic supplementary material.

Supplementary file1 (PDF 616 kb)
