# Supplementary material for: Weakly Polarized Organic Cation-Modified Hydrated Vanadium Oxides for High-Energy Efficiency Aqueous Zinc-Ion Batteries
Source: Nanomicro Lett. 2024 Feb 22;16:129. doi: 10.1007/s40820-024-01339-y (PMC10884394; doi:10.1007/s40820-024-01339-y)
Supplement: Supplementary file 1 — Supplementary file1 (PDF 616 kb) [file 40820_2024_1339_MOESM1_ESM.pdf]

Supporting Information for

Weakly Polarized Organic Cation-Modified Hydrated Vanadium Oxides for High-Energy Efficiency Aqueous Zinc-Ion Batteries

Xiaoxiao Jia[1], Chaofeng Liu[2], Zhi Wang[1], Di Huang[1], and Guozhong Cao[1, *]

[1]Department of Materials Science and Engineering, University of Washington, Seattle, WA 98195, USA

[2]School of Materials Science and Engineering, Tongji University, Shanghai, 201804, P. R. China

*Corresponding author. E-mail: gzcao@uw.edu (Guozhong Cao)

S1 Supplementary Figures

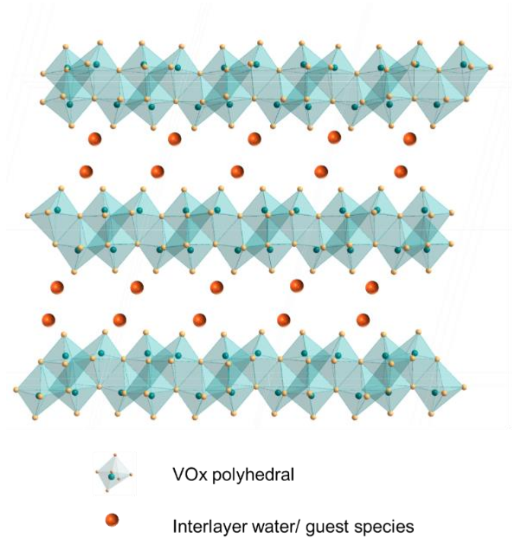

Fig. S1 A schematic illustration of the $M_xV_8O_{20}\cdot nH_2O$ phase

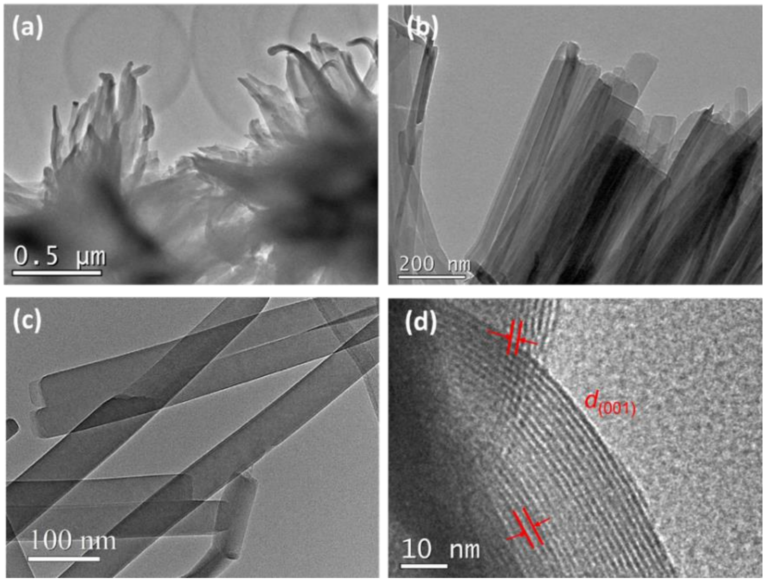

Fig. S2 TEM images of TMPA-VOH

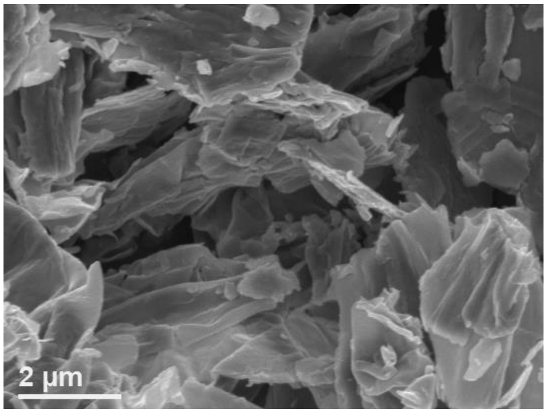

Fig. S3 SEM image of VOH

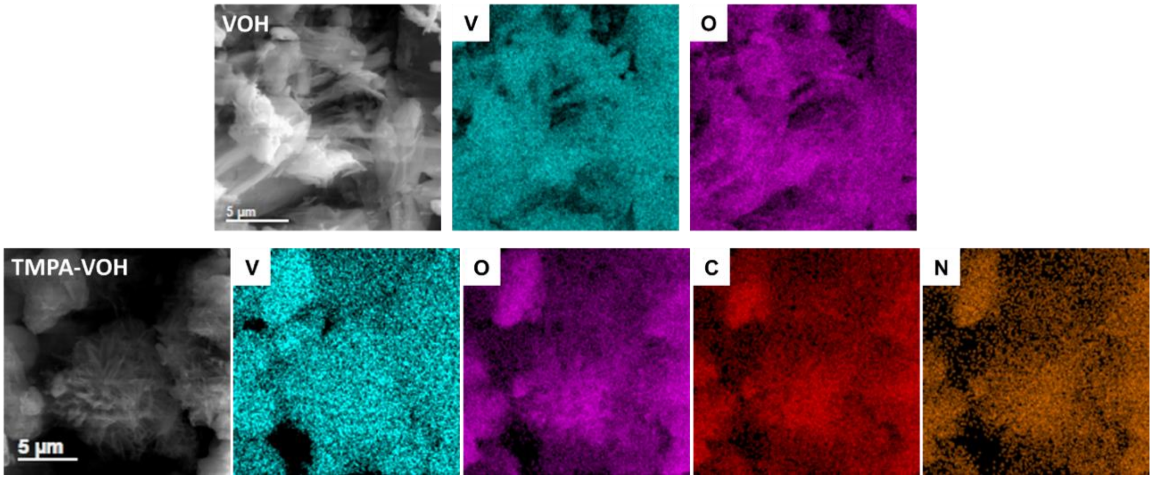

Fig. S4 SEM-EDS mapping of TMPA-VOH and VOH

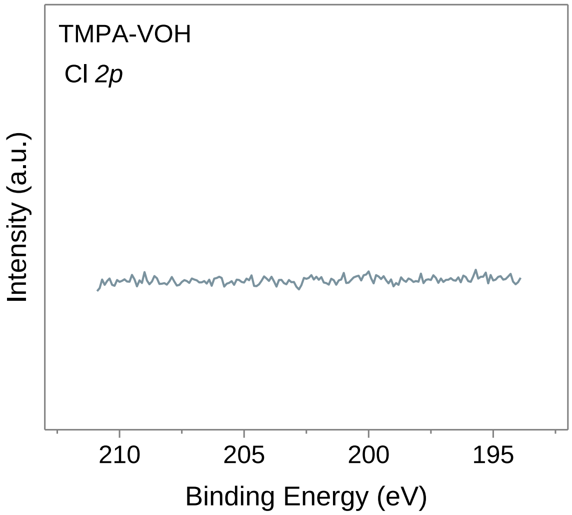

Fig. S5 XPS Cl *2p* region of TMPA-VOH

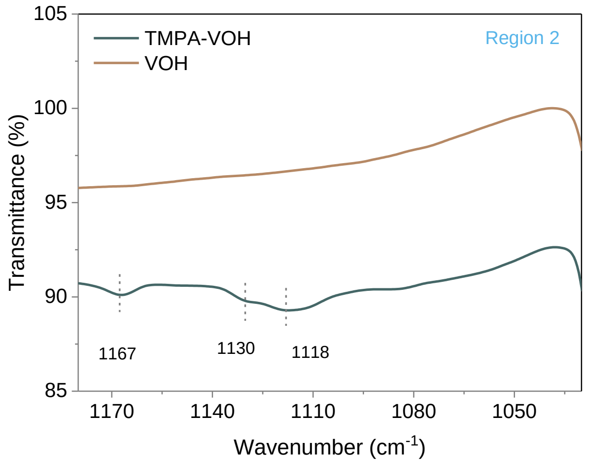

Fig. S6 Enlarged FTIR spectra of TMPA-VOH and VOH in the region of 1180-1030 cm^{-1}

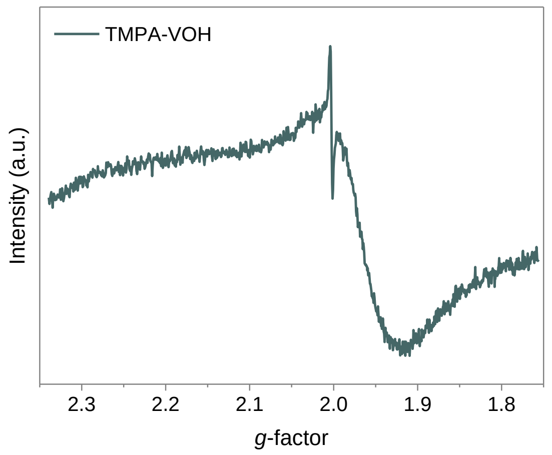

Fig. S7 EPR spectra of TMPA-VOH

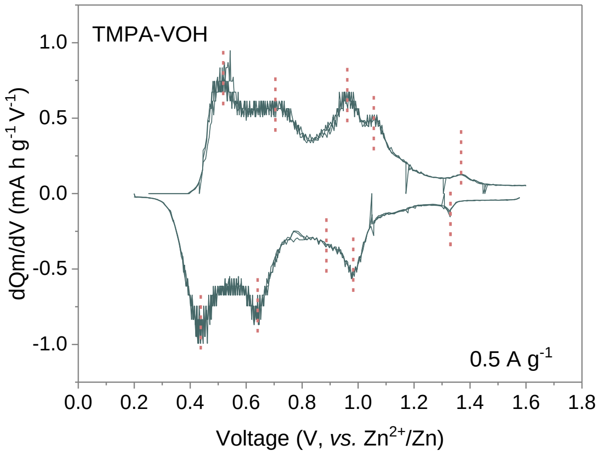

Fig. S8 The dQm/dV vs. voltage profiles of TMPA-VOH

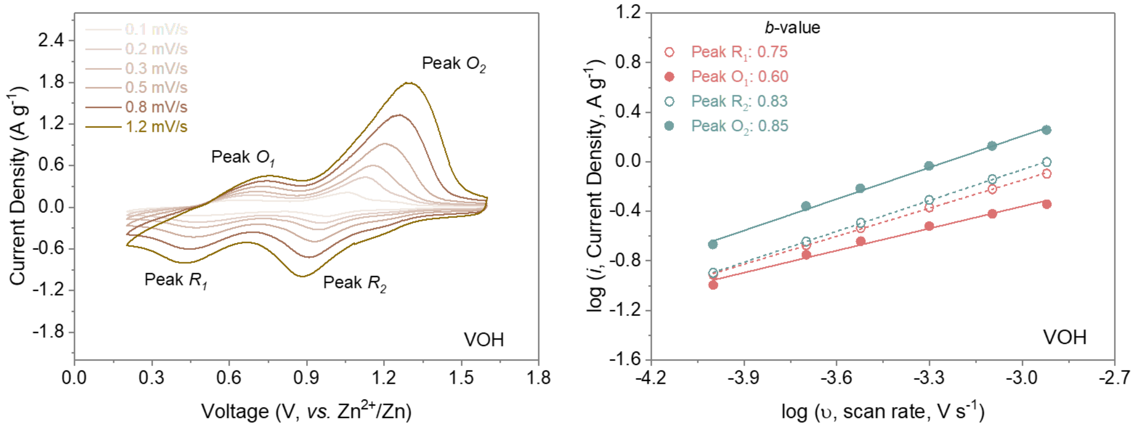

Fig. S9 (**a**) CV curves of VOH at different scan rates. (**b**) *b*-values of each redox peak in CV curves for VOH

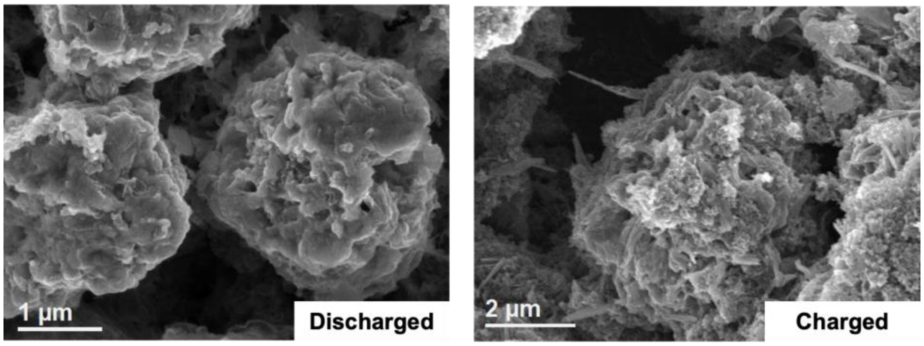

Fig. S10 The *ex situ* SEM images of TMPA-VOH electrodes at fully discharged and charged state

S2 Supplementary Tables

Table S1 Frequencies and assignment of the FTIR bands of TMPA-VOH and VOH

**Wavenumber/cm^{-1}**		**Assignment (vibration mode)**
**TMPA-VOH**	**VOH**	
742	738	Asymmetric stretching of V-O-V
770	—	Out-of-plane ring deformation
841	—	N-CH_3 symmetric stretching + (N-C) stretching +ring (C-H) stretching
944	—	Out-of-plane ring (C-H) bending+CH_2 twisting
954	—	(N-CH_3) streching+CH_3 rocking
975	less obvious	Stretching of V^{4+}=O

1007	less obvious	Stretching of V^{5+}=O
spitted	1010 (broad)	Stretching of V=O
1118	—	In-plane ring (C-H) bending+ (N-C) stretching+ CH_3 rocking
1130	—	CH_3 rocking
1167	—	In-plane ring (C-H) bending
1229	—	N-CH_3 asymmetric stretching
1298	—	In-plane ring (CCC) asymmetric stretching + CH_2 wagging
1409	—	CH_3 symmetric bending
1457	—	CH_3 asymmetric bending + In-plane ring (C-H) bending
1485	—	In-plane ring (C-H)+ CH_3 asymmetric bending
1496	—	CH_3 scissoring + In-plane ring (C-H) bending
	1613	H-O-H bending of water molecules
1632	—	C-H bending
—	3565	O-H stretching of water molecules

Table S2 Peak positions and potential gaps between each redox pair of TMPA-VOH and VOH

Sample	Peak Voltages (V)	Redox Pairs	Peak Separation (V)
TMPA-VOH	0.98/0.99	V^{5+}/V^{4+}	0.01
	0.91/1.06		0.15
	0.61/0.67	V^{4+}/V^{3+}	0.06
	0.43/0.51		0.08
	1.33/1.35		0.02
VOH	0.98/1.06	V^{5+}/V^{4+}	0.08
	0.45/0.65	V^{4+}/V^{3+}	0.20

S3 Supplementary Calculations

S3.1 Kinetics analysis from CV tests

In CV tests, the total current can be interpreted as a sum of current response from two process: a slow diffusion-controlled process (i_{diff}) and a fast surface-controlled capacitive process (i_{cap}), based on the following empirical equation [S1]:

$$i(v) = i_{cap} + i_{diff} = av^b \quad \textbf{(S1)}$$

$$\log i(v) = \log a + b \log v \quad \textbf{(S2)}$$

where both a and b are adjustable parameters. b-value (in the range of 0.5-1.0) can be determined from the slope of the plot of log i vs. log v. A b-value of 0.5 represents a slow diffusion-controlled intercalation process, while a b-value of 1.0 indicates a fast surface-controlled capacitive process. Generally, the larger the b value is, the larger the contribution from capacitive process.

Notice that the capacitive-controlled current varies linearly with the sweep rate v, while the diffusion-controlled current obeys a linear relationship with $v^{1/2}$. So, the contribution of each component can be determined based on the following equations [S1]:

$$i\ (v) = \ i_{cap} + i_{diff} = k_1 v + k_2 v^{\frac{1}{2}} \quad \textbf{(S3)}$$

$$i\ (v)/v^{\frac{1}{2}} = \ k_1 v^{\frac{1}{2}} + k_2 \quad \textbf{(S4)}$$

By plotting $i\ (v)/v^{\frac{1}{2}}$ vs. $v^{\frac{1}{2}}$, constants k_1 and k_2 can be evaluated from the slope and intercept of the line. Therefore, the contributions from the capacitive effect (k_1v) can be quantitively differentiate from diffusion-controlled processes ($k_2v^{1/2}$).

Then, the average charge storage can be estimated by:

$$Q = \frac{\int i(E)dE}{2mv} \quad \textbf{(S5)}$$

Where Q is the average charge during the charge/discharge process (C/g), m is the mass of the active material (g), i is the current response (A), v is the scan rate (V s^{-1}). Then the diffusion-controlled and capacitive controlled charge storage can be quantified.

S3.2 Diffusion coefficient and internal resistance analysis from GITT

The chemical diffusion coefficient of Zn^{2+} ions ($D_{Zn}{}^{2+}$) in cathode materials can be calculated by [S2]:

$$D_{Zn^{2+}}^{GITT} = \frac{4}{\pi\tau}\left(\frac{m \cdot V_M}{M \cdot S}\right)^2 \left(\frac{\Delta E_s}{\Delta E_\tau}\right)^2 \quad (\tau \ll \frac{L^2}{D}) \quad \textbf{(S6)}$$

Where τ (s) is the current pulse duration time; m, M, and V_M are the mass (g), atomic weight (g mol^{-1}), and molar volume (cm^3 mol^{-1}) of the active materials, respectively; S (cm^2) is the contact area of the electrode/electrolyte interface (0.785 cm^2); ΔE_s (V) is the steady state voltage change; ΔE_τ (V) is the total voltage change during a current pulse, neglecting the IR-drop.

The internal resistance (Ω) is calculated from the IR drop by [S3]:

$$\text{Internal resistance}(\Omega) = \frac{IR\ drop}{i} \quad \textbf{(S7)}$$

Where i is the applied constant current during pulses(A), and IR (V) is illustrated as below:

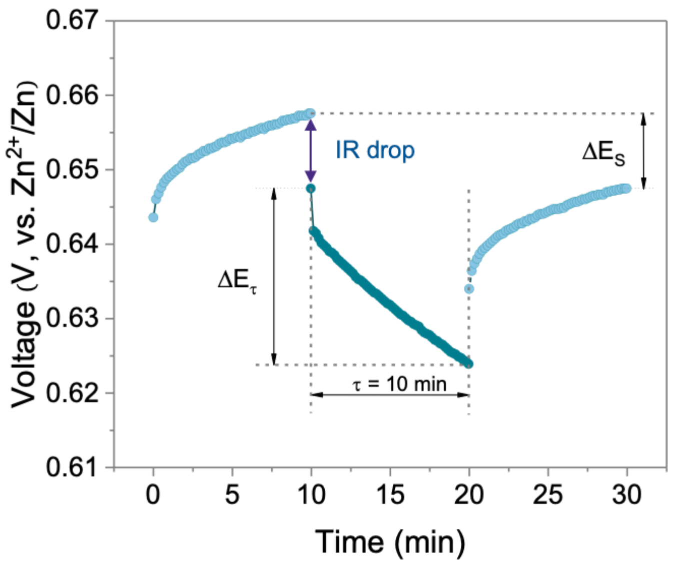

Fig. S11 Schematic illustration of ΔE_τ, ΔE_s, and IR drop

Supplementary References

[S1] J. Liu, J. Wang, C. Xu, H. Jiang, C. Li, et al., Advanced Energy Storage Devices: Basic Principles, Analytical Methods, and Rational Materials Design. Adv. Sci. **5**, 1700322 (2018). https://doi.org/10.1002/advs.201700322

[S2] W. Weppner and R. A. Huggins, Determination of the Kinetic Parameters of Mixed-Conducting Electrodes and Application to the System Li_3Sb. J. Electrochem. Soc. **124**, 1569–1578 (1977). https://doi.org/10.1149/1.2133112

[S3] M. Kemeny, P. Ondrejka, and M. Mikolasek, Comprehensive Degradation Analysis of NCA Li-Ion Batteries via Methods of Electrochemical Characterisation for Various Stress-Inducing Scenarios. Batteries **9**, (2023). https://doi.org/10.3390/batteries9010033
